# Knowledge mapping of energy research in the hospitality industry to evaluation research collaboration

**DOI:** 10.1016/j.heliyon.2023.e16591

**Published:** 2023-05-26

**Authors:** Xiumei Xu, Kunmei Liu

**Affiliations:** a School of Humanities and Social Sciences, Macao Polytechnic University, Macao 999078, China; b School of Education, Tibet University, Lhasa 850000, China

**Keywords:** Research collaboration, Cooperation network, Bibliometrics, Visualization, Hospitality, Energy

## Abstract

Analyzing collaborations on energy research in the hotel industry has important implications for promoting the research performance in this field. The Web of Science Core Collection from 1984 to 2022 was used to analyze the research contributions and cooperation networks and clusters at three levels: macro (national level), meso (institutional level), and micro (key authors and papers) using a bibliometric approach. The results show the following. (1) The cooperative relationship is the closest between China and the United States. Developed countries in Europe exhibit more academic cooperation. (2) There is a significant regional imbalance in the cooperation between universities. Leading universities rely on their strengths in energy research or hotel management and are often highly productive institutions. (3) The breadth of the authors' cooperation is insufficient. Collaborative research dominated by productive authors tends to focus on practical issues in the local hotel industry. The collaboration between experts from different disciplines benefits from the complementary advantages of these experts. (4) Hotel energy research has evolved from single-disciplinary research in the early days to interdisciplinary research in recent years. This paper provides visualizations of current conditions and deficiencies in existing research collaborations and provides a reference for analyzing the potential of research cooperation.

## 1 Introduction

The hospitality sector is the most energy-intensive part of the tourism industry after the transportation sector [1,2]. Hotel buildings are public buildings with high energy consumption due to heating, cooling, lighting, cooking, cleaning, entertainment, and other factors. The hotel industry in most countries and regions globally tends to focus on high-end and luxury features [3], resulting in high investment, high consumption, and high pollution of high-star hotels [4,5]. For example, the annual average energy utilization rates of high-star hotels in Singapore and Portugal are 427 kWh/(m^2^·[6] and 446 kWh/(m^2^·) [7], respectively, which is 20 times the energy consumption of residential buildings. In Greece, the average energy consumption per meal and bath is 5.5 kWh and 1.66 kWh, respectively [8]. In Ottawa, the annual average energy intensity of hotels is as high as 612 kWh/(m^2^·[9]. Sheng et al. (2018) found that luxury hotels in China consume on average four times more energy than other commercial buildings, highlighting the pressing need for energy-efficient operations in this sector [5]. This has prompted scholars from various research fields to delve into the issue of hotel energy consumption. For instance, Shao et al. (2020) conducted a study on hotel building energy consumption by developing a support vector machine energy consumption prediction model [10], while Todorović et al. (2020) explored the subject system in bivalent operation through the basic and advanced configurations of a heat pump system assisted with a gas boiler [11].

The rapid development of the hotel industry has caused a nonlinear growth in energy consumption, especially fossil energy consumption, and increased localized greenhouse gas emissions, which have negative impacts on the climate, environment, and economic growth. For instance, Oluseyi et al. (2016) found a strong correlation between energy consumption per unit room and CO_2_ emission levels in the hotel industry [12]. Moreover, Sofer & Potchter (2006) pointed out that the urban heat island effect is more significant around the dense hotel belt than in other areas [13].

Various countries and regions have taken measures to reduce the hotel industry's excessive dependence on energy and alleviate the resulting environmental pressure. For instance, low-carbon hotels and green hotels have been advocated by and established in many countries [14,15]. In academia, researchers have been advocating energy conservation, emission reduction, and green performance in the hotel business since the 1970s. Although the specific relationship between the total carbon emissions and the performance of hotels is unclear, there is no denying that a relationship exists [16]. In this context, the concept of green consumption has been increasingly endorsed by tourists [17,18].

Most research has focused on the following aspects. (1) The composition of hotel energy and major energy consumption sectors [[19], [20], [21]]. (2) Factors affecting hotel energy consumption [22,23]. (3) Establishing a benchmark for hotel energy consumption [[24], [25], [26]]. Early studies on hotel energy consumption were mostly case studies to demonstrate the performance of buildings and the technical level of the equipment. In recent years, an increasing number of studies have shown that interdisciplinary research has become the main research trend. The existing studies provide evidence of international cooperation in the area of energy efficiency technologies for hotel industry Furthermore, interdisciplinary studies in energy research in the hotel industry combine knowledge from various fields including engineering [27,28], environmental science [29], business and economics [22,[30], [31], [32]]. The aim is to find ways to reduce the energy consumption in hotels and increase their energy efficiency.

Collaboration is essential for energy research in the hospitality industry as it allows researchers and practitioners to combine their expertise and resources to solve complex energy-related issues. Collaboration promotes the sharing of information and best practices, leading to new insights that can be applied to real-world problems. Research collaborations assure in-depth investigations, resulting in robust findings with broader applications. Evaluating research collaborations by analyzing the knowledge mapping of energy research in hospitality is vital to understand the current state, identify knowledge gaps, and focus efforts on the most pressing issues. Assessing research collaborations helps researchers identify best practices and challenges, ultimately enhancing collaboration and research quality.

Interdisciplinary research drives innovation. Interdisciplinary research refers to the collaboration of researchers across different fields of study to solve complex problems that cannot be solved using a single discipline [33]. This collaboration brings together perspectives, methods, and theories from various disciplines, which leads to innovation in research and the development of new ideas, technologies and approaches [34,35]. Since research is a complex social undertaking, it relies on collaboration between researchers. Furthermore, cooperation has a substantial influence on knowledge exchange and innovation. Research cooperation has become an important aspect of bibliometrics due to an increase in bibliometric studies [36]. However, there are very few reports on collaborative research on hotel energy-related issues. Therefore, this paper uses bibliometric and visualization methods to explore research cooperation among countries, institutions, and authors to understand the current organization of academic activities in energy research in the hotel industry. The citation networks of highly cited papers were also analyzed to clarify how knowledge is transferred and developed.

The remainder of this paper is organized as follows. The methodology and data are described in Section 2. Section 3 presents the results and discussions. The conclusions are drawn in Section 4.

## 2 Methodology

### 2.1 Analytical method and software

The bibliometric approach is used to visualize the results obtained from a large number of documents to investigate the cooperation among countries, institutions, and authors and how this cooperation affects the direction of future studies. The bibliometric method is supported by mathematics and statistics and relies on measurement software to collect, process, and organize data to analyze and evaluate large amounts of literature data and make predictions. The commonly used bibliometric and visualization software packages include Bibexcel, CiteSpace, VOSviewer, and UCINET.

The software used in this paper includes CiteSpace 5.8. R3, HistCite Pro 2.0, and Scimago Graphica. Cluster analysis, centrality analysis, and burst analysis are implemented in CiteSpace 5.8. R3. The research contribution tables and citation network graphs are generated with HistCite Pro 2.0. The cooperation network between countries (publications) is created using Scimago Graphica. CiteSpace 5.8. R3 is used to explore potential knowledge and information in the literature. This software is a multivariate and dynamic citation visualization software used in scientometrics [37]. It has been widely used for text analysis and data mining of bibliographic databases [38].

### 2.2 Data sources and screening

**Fig. 1:**
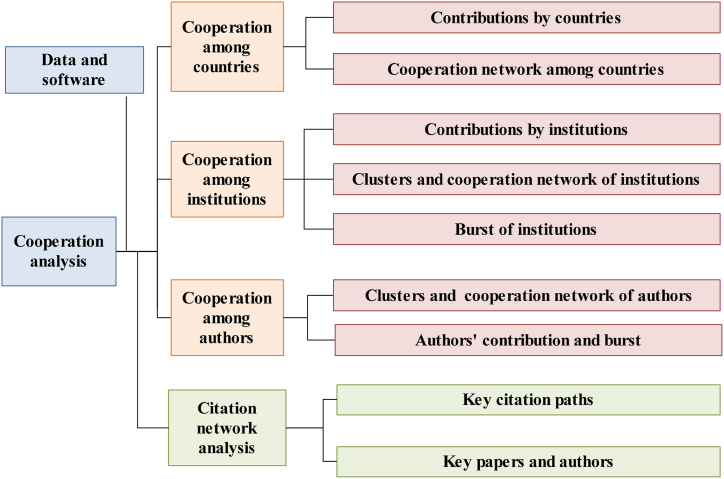
The framework of this study.

We use the topic retrieval method to ensure the comprehensiveness of the literature data. This Web of Science (WoS) Core Collection is used as the data source, and literature searches are conducted with the topics “hotel & energy”, or “hospitality & energy”, or “accommodation & energy”, or “hotel & power” or “hotel & diesel oil”, and the retrieval time is March 6, 2022. After removing duplicates from the original 1362 documents, 996 English “article type” documents were obtained. The research framework of this paper is shown in Fig. 1.

### 2.3 Research limitations

**Fig. 2:**
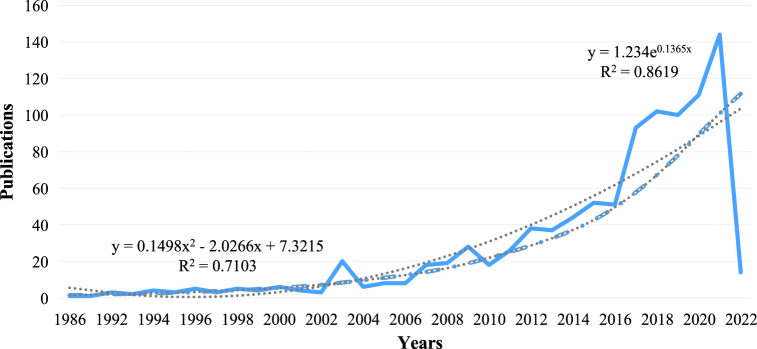
Annual trend of the publications and citations.

Due to the utilization of three visualization software, namely CiteSpace 5.8. R3, HistCite Pro 2.0, and Scimago Graphica in this paper, some of the figures and tables may have different starting years than the original data, as the parameters were adjusted according to research needs during the drawing process. For example, Fig. 2 was drawn based on the publication and citation frequency of literature processed by HistCite Pro 2.0. Table 5 shows the burst of institutions based on the original data processed by CiteSpace 5.8. R3, while Table 7 shows the burst of authors based on the original data processed by CiteSpace 5.8. R3. Finally, Fig. 11 was visualized based on the citation network of the top 25 highly cited papers in the original data processed by HistCite Pro 2.0.

## 3 Results and discussion

### 3.1 Overall status of the publications

The annual trend of the publication volume and the citations of the literature on hotel energy-related fields is shown in Fig. 2.

Before 2002, the number of publications and citations in the literature was very low. After 2007, the growth rate of publications started to increase. From 2017 to the present, the annual publication volume has remained high. During this period, the number of citations also exhibited rapid growth. Since the data were retrieved in the first half of 2022, only a few documents existed in that year. However, this does not affect our evaluation of the overall trend.

### 3.2 Cooperation among countries

#### 3.2.1 Contributions by countries

**Fig. 3:**
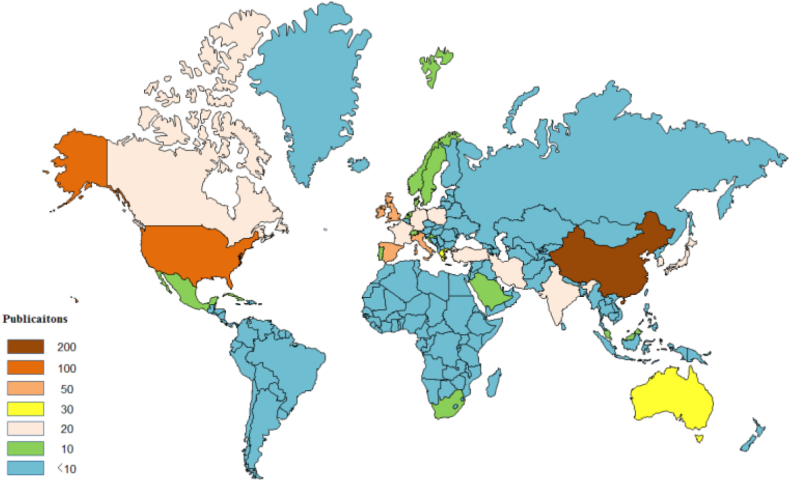
Number of publications by countries.

There are 89 countries that have published related papers. Fig. 3 shows the number of publications in different countries globally. Further, Table 1 lists 16 countries that produced more than 20 publications. The top 5 countries are China, USA, UK, Italy, and Spain. China produced 233 papers. The contributions of the top five countries account for 55.7% of all publications in the other countries. The 16 countries listed in Table 1 are located in various parts of the world and include developing and developed countries. Therefore, the energy consumption in the hotel industry has attracted worldwide attention.

**Table 1:** The top 16 countries based on the number of publications.

No.	Country	Recs	TLCS	TGCS
1	China	233	474	5630
2	USA	123	221	3733
3	UK	73	184	2156
4	Italy	63	98	1524
5	Spain	63	91	1074
6	Australia	43	249	1394
7	Greece	36	193	1027
8	Japan	29	21	970
9	Turkey	28	80	657
10	Germany	26	9	449
11	India	26	15	297
12	Canada	23	44	645
13	South Korea	22	20	475
14	France	21	15	400
15	Iran	21	28	385
16	Poland	20	14	223

Note: Recs, TLCS, and TGCS represent “records”, “total local citation score”, and “total global citation score”, respectively (similarly hereafter).

#### 3.2.2 Cooperation networks among countries

**Fig. 4:**
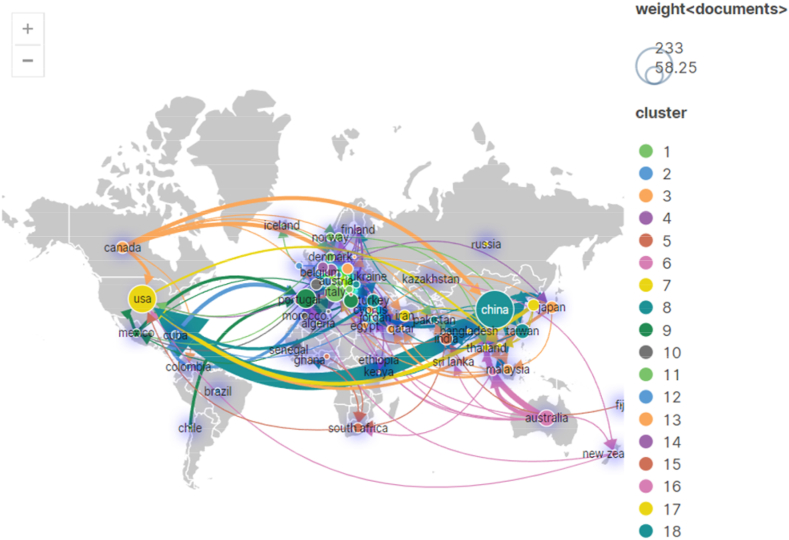
Cooperation network among countries (publications).

The relationship based on research cooperation between countries is further visualized. The cooperative relationship among countries is mapped in Fig. 4. The size of the nodes and the thickness of the lines indicate the country's importance in the cooperation network and the cooperation level between the country and other countries, respectively. For example, China and the United States have the two largest nodes and the thickest connection lines, indicating that the two countries have the largest number of collaborative papers and the highest level of cooperation. In addition, cooperation between countries with similar cultures in the same region is frequent; for example, the level of research cooperation within Europe is high. However, the results show that research in hotel energy is not limited to countries with geographical proximity, and transcontinental collaboration is occurring.

**Fig. 5:**
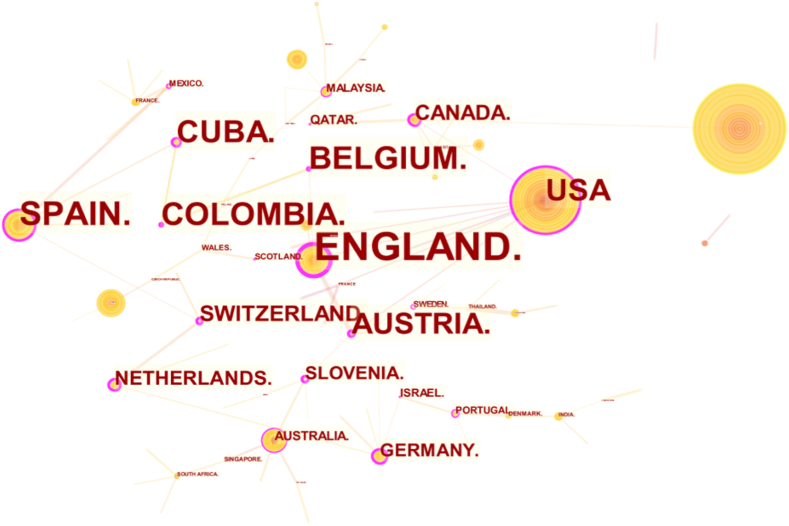
Cooperation networks between countries (centrality).

Table 2 lists the centrality of the top 17 countries, and the cooperation networks among countries based on the centrality is shown in Fig. 5. The larger the font of a node, the higher the centrality is. In bibliometrics research, centrality refers to the degree to which a given publication, author, institution or country is situated as a pivotal node within a network of scholarly communications [39,40] (S. Behara et al., 2014; Li et al., 2018). Centrality measures in bibliometrics research has been shown to have several applications, including identifying key contributors to a field of research, tracing the flow of knowledge across disciplines, and predicting future trends in scholarship [41] (Fortunato, 2010). Based on centrality, this paper evaluates the contribution of countries and institutions in the cooperation network.

**Table 2:** The top 17 countries with centralities over 0.10

No.	Countries	Centrality	Count	Year	No.	Countries	Centrality	Count	Year
1	ENGLAND	0.47	57	2008	10	NETHERLANDS	0.23	18	2009
2	SPAIN	0.40	58	2009	11	SLOVENIA	0.22	8	2009
3	BELGIUM	0.38	8	2012	12	GERMANY	0.21	23	2009
4	COLOMBIA	0.38	7	2016	13	AUSTRALIA	0.16	38	2008
5	CUBA	0.37	11	2011	14	QATAR	0.16	5	2014
6	USA	0.36	124	1995	15	MALAYSIA	0.14	15	2009
7	AUSTRIA	0.35	9	2008	16	ISRAEL	0.14	2	2017
8	CANADA	0.26	22	2009	17	PORTUGAL	0.12	15	2009
9	SWITZERLAND	0.26	13	2013

With the help of CiteSpace software, the cooperation network between countries has been visualized, as shown in Fig. 5. If a node's centrality degree in the network is large, this node has more control over the flow of resources, and more nodes are needed to communicate with each other through this node [42]. England, Spain, Belgium, and Colombia have high centrality values, exceeding 0.38. Thus, these countries have a strong ability to control academic resources and coordinate with other countries in the cooperation network. According to Fig. 5, the cooperation between England and USA promotes the influence of Canada and European countries, such as Austria. In addition, Spain, Switzerland, and the Netherlands have become hubs connecting some European countries for research cooperation. Meanwhile, Spain and Belgium are also key nodes; thus, these countries strengthen the cooperation between South American countries and European countries.

However, Table 2 shows that no countries have very high centrality, indicating that no country has a monopoly in the cooperation network. Therefore, other countries have more room to choose their partners in the network.

### 3.3 Cooperation among institutions

#### 3.3.1 Contributions by institutions

The data contain 1218 institutions and 1850 institutions with subdivisions. Table 3 lists the institutions that have published more than 7 papers. The research output of Hong Kong Polytechnic University is the largest, far exceeding that of Tianjin University, which ranks second. Most of the institutes in Table 3 are located in China. Universities are the dominant type of research institution in the hotel energy research field.

**Table 3:** Institutions with more than 7 publications.

No.	Institution	Recs	TLCS	TGCS
1	Hong Kong Polytech Univ	46	188	1256
2	Tianjin Univ	17	67	305
3	City Univ Hong Kong	15	48	401
4	Tongji Univ	14	13	339
5	Griffith Univ	13	64	290
6	Tsinghua Univ	13	13	399
7	Shanghai Jiao Tong Univ	12	38	409
8	Univ Palermo	11	60	504
9	Univ West London	10	3	85
10	Southeast Univ	9	2	115
11	Chongqing Univ	8	16	173
12	Dalian Univ Technol	8	21	216
13	Islamic Azad Univ	7	12	203
14	Jinwen Univ Sci & Technol	7	35	165
15	Natl Tech Univ Athens	7	18	172
16	Natl Univ Singapore	7	75	246
17	North China Elect Power Univ	7	3	72
18	Univ Moratuwa	7	2	29

#### 3.3.2 Clusters and cooperation network of institutions

**Fig. 6:**
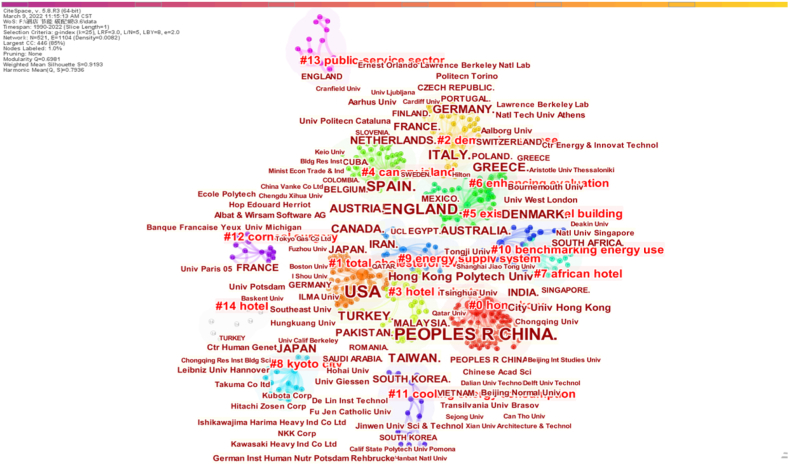
Dual-map overlay of institutional cooperation.

The dual-map overlay of the institutional cooperation and clusters of countries in hotel energy research is shown in Fig. 6. The dual-map overlay is a visualization feature provided by the bibliometric analysis software CiteSpace. This feature enables the comparison of two maps generated from two different time periods or data sets [43](Chen, 2017). The maps are overlaid on top of each other, with the nodes and links colored according to their presence or absence in each of the maps [44](Chen, 2006). This allows researchers to identify the nodes and links that are unique to each map, as well as those that are common to both maps.

**Fig. 7:**
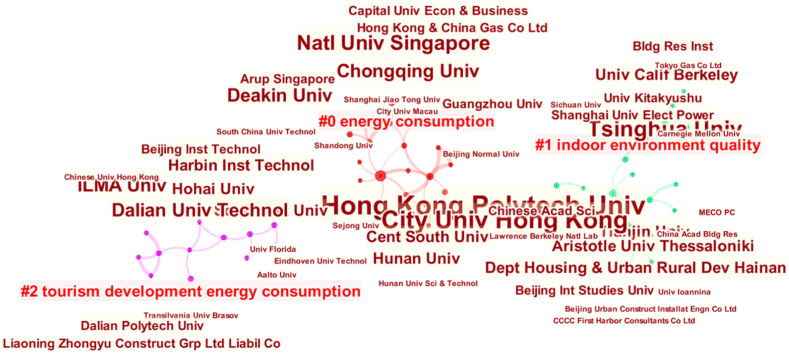
Cooperation networks among institutions.

The institutions in different regions have different research focuses. For example, the label of Cluster #0 is “Hong Kong”, indicating that these institutions aim to solve the energy problems of local hotels. Similar observations are made for research institutions in Japan and Africa. Three major institutional cooperative clusters are extracted and shown in Fig. 7.

First, the data in the red cluster (Fig. 7) show that Hong Kong Polytechnic University and City University of Hong Kong publish more papers and have higher centrality than other institutions, as shown in Table 4. Hong Kong Polytechnic University published two papers in this field in 2000, indicating that this university focused on this topic early. Hunan University, Chongqing University, and Macao City University have research cooperations with these two universities. In addition, there are also research cooperation relationships among the National University of Singapore, Shanghai Jiaotong University, Guangzhou University, Chongqing University, and Deakin University. Deakin University and Chongqing University have intermediary roles in the network. These institutions in the red cluster mainly focus on energy consumption and the study of different fields.

**Table 4:** The top 18 institutions with centralities over 0.01

No.	Institution	Centrality	No.	Institution	Centrality
1	Hong Kong Polytech Univ	0.04	10	Chongqing Univ	0.01
2	City Univ Hong Kong	0.04	11	Dalian Univ Technol	0.01
3	Tsinghua Univ	0.04	12	Aristotle Univ Thessaloniki	0.01
4	Chinese Acad Sci	0.03	13	Shanghai Univ Elect Power	0.01
5	Harbin Inst Technol	0.03	14	Deakin Univ	0.01
6	Beijing Int Studies Univ	0.03	15	Univ Kitakyushu	0.01
7	Southeast Univ	0.02	16	Univ Calif Berkeley	0.01
8	Hohai Univ	0.02	17	Dept Housing & Urban Rural Dev Hainan	0.01
9	Tianjin Univ	0.01	18	ILMA Univ	0.01

Second, in the green cluster, three small clusters have formed around Tsinghua University, Tianjin University, and Aristotle University Thessaloniki. These institutions focus primarily on indoor environmental quality, and the research started later than in the red cluster.

Finally, institutions in the purple cluster mainly focus on hotel energy consumption against the background of tourism development. Harbin Institute of Technology, Southeast University, Hohai University, and Dalian University of Technology are intermediaries in this cluster. Institutions in this cluster started research later than those in the other two main clusters, and the number of publications is relatively low.

#### 3.3.3 Citation bursts of institutions

Citation bursts of institutions based on CiteSpace refer to the sudden and significant increase in the number of citations received by an institution or group of papers within a specific period [45] (Chen et al., 2012). The goal of citation bursts analysis is to identify areas of research that are gaining momentum and to determine the institutions and scholars that are leading the way in those areas [46](Che et al., 2022). The role of citation bursts analysis is to provide insight into the evolution of research topics and to help identify new research areas. This analysis can be used by scholars, researchers, and funding agencies to discover new research frontiers and to make informed decisions about funding priorities. Additionally, it can help academic institutions evaluate their research strengths and areas where they may need to concentrate more effort.

Citespace 5.8. R3 software was used for citation burst detection of major international research institutions in hotel energy-related fields from 1990 to 2022 (γ = 0.6). The results are listed in Table 5. There are 12 institutions, of which 11 institutions are universities and one is the CIBSE (Royal Society of Building Service Engineers). The burst value of North China Electric Power University is the highest (3.8), and the burst period is 2010–2013. J. J. Wang is the author with the highest burst value at this institution, and most of the publications [[47], [48], [49]] were published in 2010–2011, indicating that the contribution of this institution in the field of hotel energy is largely attributed to this author.

**Table 5:**
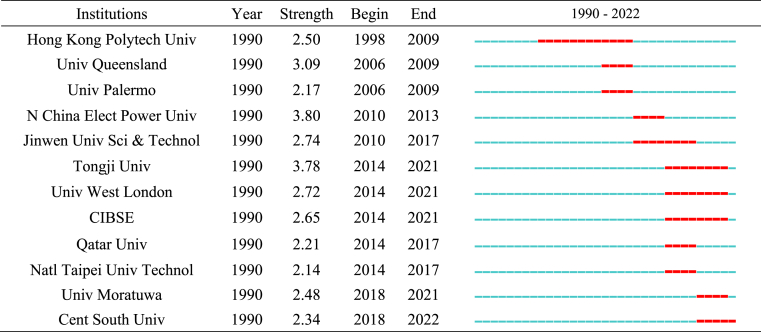
Top 12 Institutions with the strongest citation bursts.

Hong Kong Polytech University had the longest burst period of 11 years (1998–2009). During this period, Yu published the most papers [[50], [51], [52], [53], [54]], demonstrating that this author contributed largely to the high burst value. In recent years, the University of Moratuwa and Central South University have emerged as institutions with high burst values. Research in this field started in 2018 at these two institutions. We analyzed the authors' cooperative network to clarify which authors have made outstanding contributions and their cooperative relationships.

### 3.4 Cooperation among authors

#### 3.4.1 Clusters and cooperation network of authors

**Fig. 8:**
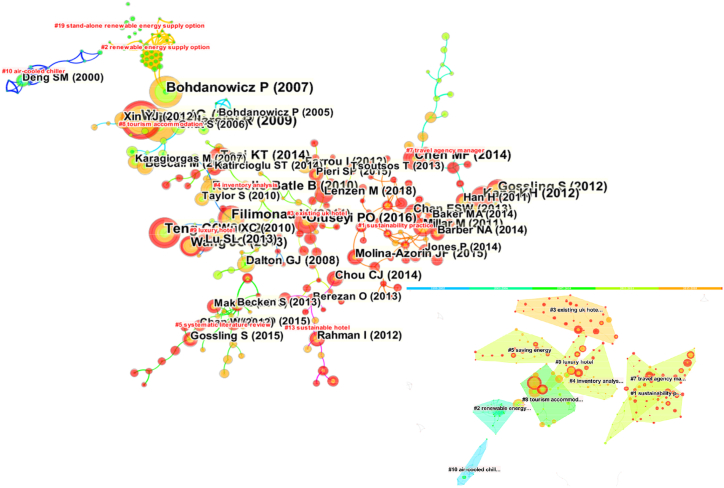
Dual-map overlay of the authors' cooperation.

The dual-map overlay of the author clusters and the authors' collaboration networks is shown in Fig. 8.

The 9 large clusters in Fig. 8 focus on the following topics: (1) energy conservation and sustainable development in the hotel industry (e.g., Cluster #1 and Cluster #5); (2) energy consumption of high-star hotels in specific areas (e.g., Cluster #3 and Cluster #9); (3) energy savings of hotel equipment and renewable energy options (e.g., Cluster #2, Cluster #4 and Cluster #10); (4) hotel energy consumption management (e.g., Cluster #6 and Cluster #7). These clusters involve a wide range of fields, indicating that hotel energy-related topics have aroused the interest of many scholars, and interdisciplinary research is increasing in academia. However, it is worth noting that the connections within these clusters are not dense, suggesting there is much room for cooperation and communication among scholars.

**Fig. 9:**
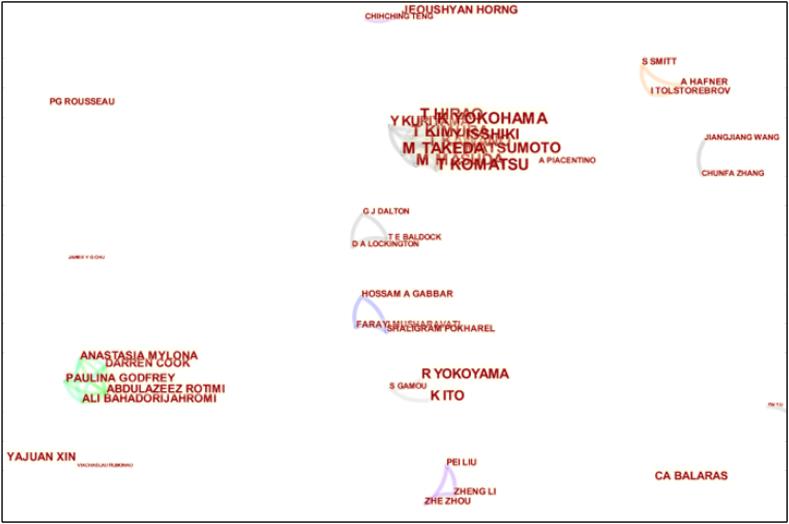
Authors' cooperation network.

The authors' collaboration networks are shown in Fig. 9. Authors with larger fonts have a higher research output and vice versa. The results suggest that the collaboration among authors is not ideal, and there is a lack of large network structures. There are only two sub-networks with favorable network characteristics, indicating that many productive authors do not collaborate with other scholars.

#### 3.4.2 Authors' contributions and bursts

Among the 2842 authors, 31 authors published more than 5 papers. The information on these 31 authors is listed in Table 6.

**Table 6:** Top 31 authors based on the number of publications.

No.	Author	Recs	TLCS	TGCS
1	Bahadori-Jahromi A	10	3	85
2	Cook D	10	3	85
3	Mylona A	10	3	85
4	Becken S	9	83	296
5	Godfrey P	9	3	82
6	Zhu N	9	24	210
7	Wang JJ	8	40	487
8	Chan WW	7	43	198
9	Filimonau V	7	35	137
10	Wang X	7	0	87
11	Byrne P	6	14	197
12	Horng JS	6	35	165
13	Ito K	6	3	79
14	Piacentino A	6	34	419
15	Wu W	6	38	131
16	Xu PP	6	35	275
17	Yokoyama R	6	3	79
18	Baldock TE	5	84	567
19	Cardona F	5	18	237
20	Chan KT	5	11	106
21	Chan W	5	29	94
22	Dalton GJ	5	84	567
23	Fasna MFF	5	1	6
24	Gao WJ	5	17	202
25	Gunatilake S	5	1	6
26	Li D	5	31	74
27	Lockington DA	5	84	567
28	Park S	5	0	14
29	Warren C	5	20	85
30	Yu FW	5	11	106
31	Zhai XQ	5	21	211

Bahadori-Jahromi, Cook, and Mylona are the top 3 productive authors. The top 49 authors with the strongest citation bursts (γ = 0.1) are listed in Table 7. An analysis of Table 6, Table 7 indicates that some highly productive authors are also those with the strongest citation bursts, such as Bahadori-Jahromi, Cook, Becken, Ito, Yokoyama, and Wang and Yu.

**Table 7:**
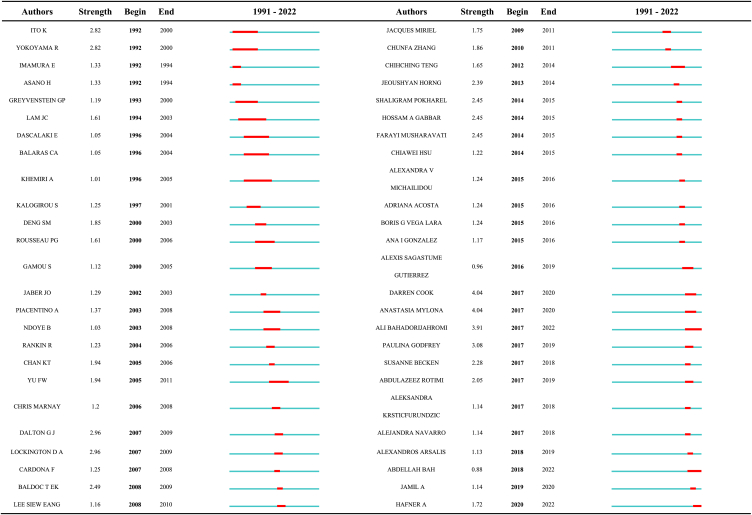
Top 49 authors with the strongest citation bursts.

**Fig. 10:**
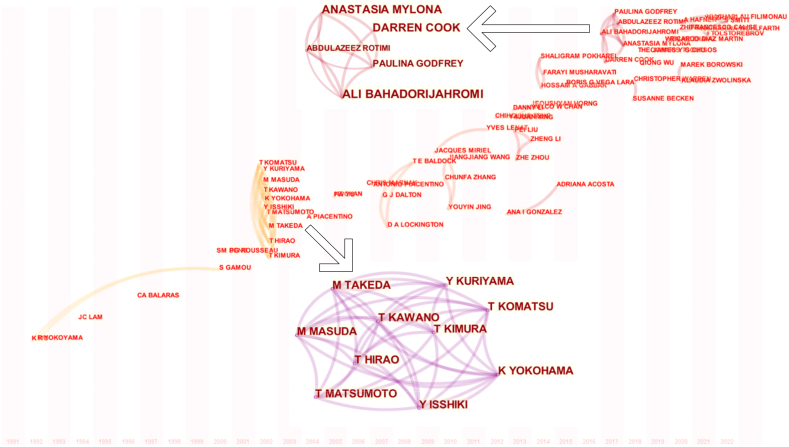
The evolution of the authors' collaborations over time.

Furthermore, the collaboration network among these authors is presented on a time zone map, as shown in Fig. 10. The timezone map in CiteSpace is a visual representation of the distribution of scientific publications across different time zones [55] (Wang et al., 2022). Its role is to help researchers understand how research activity is distributed globally and to identify potential areas of collaboration with researchers in different time zones [56] (Guo et al., 2022). By analyzing the timezone map, researchers can identify patterns, trends, and potential collaborations more easily.

Fig. 10 shows a diagram of the authors' bursts and the evolution of the authors' research in different periods. Before 2000, the author with high burst values included Yokoyama, Imamura, Ito, Lam, and Balaras. However, there was little collaboration among these authors. The bursts of Lam and Khemiri lasted for 10 years, indicating that they were the leading authors in this research stage. For example, Lam published 3 papers during this period, focusing on hotel energy consumption and management [57] and then moved to energy performance studies on hotel heat pumps [58,59].

Ito and Yokoyama have the highest burst values (2.82). Both authors published 6 papers during this period, and they collaborated on research, focusing on a cogeneration system of a hotel to reduce energy costs [60,61]. After 2001, the duration of the authors' bursts decreased. The authors with the longest duration were Rousseau and Yu (6 years). Yu is listed in Table 7, indicating his high productivity. Yu focused on hotel architecture in Hong Kong, such as chiller energy performance and electricity consumption [[50], [51], [52]]. Yu is a researcher at Hong Kong Polytechnic University, and his main co-author is Chan, who is also listed in Table 7.

After 2001, the authors' burst values improved significantly. The top three authors were Cook, Mylona, and Ali, whose bursts values were 4.04, 4.04, and 3.91, respectively. These scholars had high productivity and published 10 papers. The burst periods of the three scholars started in 2017, indicating that they concentrated on hotel energy topics in recent years. Cook, as a major co-author of Salem, focused on the energy performance and environmental impacts of British hotels, such as carbon emissions [28,62,63]. In addition, Cook, Mylona, Ali, Paulina, and Abdulazeez formed a small collaborative research network. Therefore, there is a high degree of overlap in the research fields of these scholars.

In addition, a cooperative network including the scholars Komatsu, Kimura, Kuriyama, and others was formed in 2002. This network is the most connected sub-network in the collaboration graph. However, the research output of the authors in this network is not high, and they collaborated on only one paper.

Since no close and long-term collaboration network of authors was formed, it is impossible to capture the evolution of the research content based solely on the collaboration network of authors. Therefore, it is necessary to analyze the citation relationship of highly cited papers to explore how knowledge in this field is shared among different authors from a microscopic perspective.

### 3.5 Citation path evolution

#### 3.5.1 Analysis of the citation network

The analysis of the citation relationship between papers enables the summary, refinement, and prediction of future research on hotel energy-related issues. The citing and cited relationships between papers provide information on the importance of publications [64]. A common approach is the citation path of highly cited papers [65]. The citation chronology diagram created in Histcite software can help researchers identify important papers, determine the correlation between papers, and trace the research path to understand how knowledge in this field evolved [66].

**Fig. 11:**
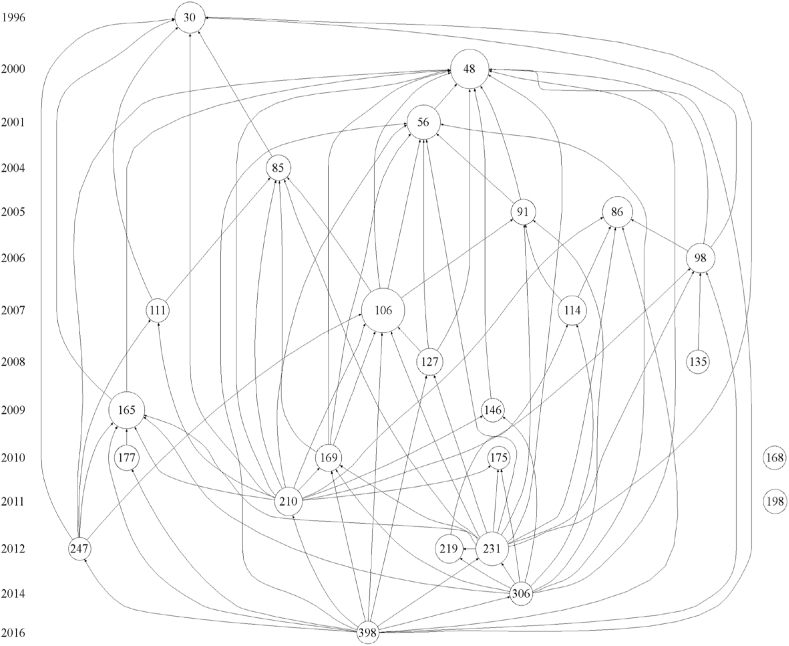
Citation chronology of hotel energy research.

The most important indicators used in the Histcite software are the local citation score (LCS) and global citation score (GCS). The LCS refers to the number of citations in the WoS database, and the GCS refers to the total number of citations globally [67]. The LCS value is more informative [68]. We imported the data into Histcite software, sorted them by LCS, and created the citation chronology diagram of the first 25 key papers (Fig. 11). The information on the nodes in the graph is listed in Table 8.

**Table 8:** Information on the citation chronology nodes.

No.	Node information (author/year of publication/journal)	LCS	GCS	Refs
30	Santamouris M, 1996, ENERG BUILDINGS, V24, P65	36	77	[69]
48	Deng SM, 2000, ENERG BUILDINGS, V31, P7	57	121	[70]
56	Becken S, 2001, ECOL ECON, V39, P371	43	143	[4]
85	Dascalaki E, 2004, ENERG BUILDINGS, V36, P1091	23	65	[71]
86	Trung DN, 2005, J CLEAN PROD, V13, P109	36	141	[72]
91	Warnken J, 2005, TOURISM MANAGE, V26, P367	23	53	[73]
98	Onut S, 2006, ENERG BUILDINGS, V38, P964	31	107	[74]
106	Bohdanowicz P, 2007, ENERG BUILDINGS, V39, P82	70	176	[75]
111	Karagiorgas M, 2007, ENERG BUILDINGS, V39, P416	21	38	[8]
114	Erdogan N, 2007, TOURISM MANAGE, V28, P604	31	183	[76]
127	Dalton GJ, 2008, RENEW ENERG, V33, P1475	27	188	[77]
135	Ali Y, 2008, ENERG CONVERS MANAGE, V49, P3391	20	44	[78]
146	Beccali M, 2009, RENEW ENERG, V34, P82	20	46	[79]
165	Priyadarsini R, 2009, ENERG BUILDINGS, V41, P1319	50	89	[6]
168	Wang JJ, 2010, APPL ENERG, V87, P1325	21	305	[48]
169	Rossello-Batle B, 2010, ENERG BUILDINGS, V42, P547	26	107	[80]
175	Taylor S, 2010, BUILD ENVIRON, V45, P1389	19	42	[81]
177	Wu XC, 2010, ENERG POLICY, V38, P4520	23	83	[1]
198	Millar M, 2011, CORNELL HOSP Q, V52, P302	22	162	[82]
210	Filimonau V, 2011, J CLEAN PROD, V19, P1917	30	106	[26]
219	Teng CC, 2012, INT J HOSP MANAG, V31, P199	29	81	[83]
231	Wang JC, 2012, ENERG BUILDINGS, V49, P268	41	77	[84]
247	Farrou I, 2012, ENERG BUILDINGS, V55, P553	19	47	[85]
306	Tsai KT, 2014, TOURISM MANAGE, V42, P13	21	73	[86]
398	Oluseyi PO, 2016, ENERG BUILDINGS, V118, P106	19	35	[12]

Fig. 11 shows the publication time and mutual citation relationship of the 25 most influential papers in hotel energy research. The publication year is shown on the y-axis, and the nodes parallel to the year represent the articles published in that year. The node size represents the citation frequency of the paper; it is proportional to the LCS value. The lines with the arrows represent the citation relationship between papers; the arrows point to the cited papers.

The citation relationship in Fig. 11 is divided into two parts. Except for the isolated Node #168 and Node #198 on the far right, the remaining 23 papers are part of the interconnected citation network, which has two stages: early independent development before 2000 and higher connection after 2000. The LCS values of the 4 top-ranking nodes (#106, #48, #165, and #231) among the 25 nodes exceed 40. These are the highest cited papers and are representative of the research in this field.

#### 3.5.2 Key citation paths

**Fig. 12:**
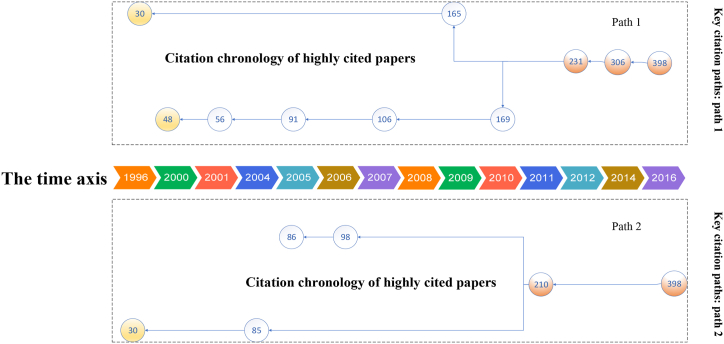
Key citation paths of hotel energy research.

We identified the key citation paths to analyze the evolution of global hotel energy research. The principles for determining the key citation paths are as follows. The starting point is the earliest publication, and the endpoint is the most recent publication. Second, the largest number of nodes in the path is used. Third, the path contains nodes with high LCS values, and the largest number of connections with other papers is used. The two key citation paths are shown in Fig. 12.

Each path has two branches. Path 1 includes 10 nodes, covering 20 years (1996 to 2016). Its two branches start at nodes #30 and #48. After more than 10 years, the two branches converge at Node #231 in 2012 and Node #306 in 2014, ending at Node #398.

One branch of Path 1 is #30 - #165, which focuses on energy-saving potential and energy improvement of hotels in specific countries or regions. The other branch is #48 - #56 - #91 - #106 - #169, which focuses on the development of benchmarks for hotel energy utilization.

In contrast, Path 2 has fewer nodes. The starting points of its two branches are #30 and #86, respectively. These two branches converge at Node #210 in 2011, ending at Node #398. One branch consists of #86 and #98. Its research focus is the quantification of hotel energy utilization efficiency. The other branch includes nodes #30 and #85, focusing on energy innovation.

The two paths have the same starting and end points. Path 1 and Path 2 have some overlap in the research content. However, Path 1 is more comprehensive in terms of the disciplines. Path 1 includes research on energy, architecture, tourism, and hotel management.

The yellow nodes in Fig. 12 are the key starting points (#30 and #48). These two papers can be regarded as groundbreaking papers. Santamouris is a Greek scholar who studied hotel energy conservation based on the energy audit of 158 hotels in Greece. In 1996, he first suggested that hotel buildings have the highest energy consumption among public buildings. This author conducted a simulation study of energy-saving strategies in the heating, cooling, and lighting sectors, revealing the potential for 20% energy savings in hotel buildings [69]. In addition, Deng & Burnett (2000) evaluated the energy performance of hotels in Hong Kong and found that the electricity consumption by air conditioning accounted for the largest proportion of total energy consumption [70].

The orange nodes denote the highly citing papers in the citation network. Therefore, these papers tend to be comprehensive studies. Nodes #231, #306, and #398 are located in the backbone of Path 1. These 3 papers reflect the research frontiers in recent years and the dominant topics. Paper #231 applies multiple regression to analyze and predict the energy use intensity of hotels [84]. In addition, Paper #306 calculates the carbon emissions caused by energy consumption in hotels and family inns and proposes countermeasures to reduce emissions [86]. The academic backgrounds of the authors (including the collaborators) of the two papers include tourism management, architecture, and leisure science, indicating that hotel energy research has become more interdisciplinary in recent years. Paper #398 uses regression analysis to explore the relationship between the energy consumption of the hotel industry and carbon emissions caused by energy consumption [12]. Meanwhile, this paper sets the benchmark of hotel energy consumption and puts forward countermeasures for carbon emission reduction. Therefore, Paper #398 reflects the integration of key papers in Path 1.

In Path 2, Paper #210 summarizes life cycle analysis studies in hotel energy consumption and emission reduction and selects two typical hotels to analyze their energy efficiency and carbon emission intensity [26]. Therefore, it can be concluded that Paper #210 is an interdisciplinary paper in the citation network. Filimonau, the first author of this paper, was active in academic research during 2011–2021, primarily focusing on energy and carbon efficiency research in the hotel industry and publishing seven papers in related fields during this period. The author's professional field is tourism management, and his co-authors' professional background includes energy engineering, industrial engineering, management, leisure, and geographical science. The collaboration of these scholars suggests that interdisciplinary research will be a major trend in the future.

### 3.6 Discussion

This paper provides visualizations of national, institutional, and author collaborations on hotel energy research. First, although hotel energy research has flourished worldwide, and some developing countries are involved. Developed countries, especially those in Europe, have advantages regarding the control of academic resources and cooperation in collaborative research networks. Furthermore, the cooperation among developed countries also influences countries in other regions. In addition, developed countries where hotel research is more specialized, such as Switzerland, have more advantages in controlling and coordinating research resources.

Second, it was found that universities are the dominant institutions, whereas the level of participation of research institutes and enterprises is very low. In particular, Hong Kong Polytechnic University has contributed the most to hotel energy research and has the highest centrality. This finding shows that Hong Kong Polytechnic University occupies an important position in the cooperation network and serves as a bridge for academic exchanges and cooperation among institutions. Hong Kong Polytechnic University is the most active academic institution in Asia because its tourism research is second to none in Asia.

The clustering results indicate that institutional collaboration has focused on indigenous issues. Hong Kong Polytechnic University has concentrated on analyzing the energy-saving potential of local hotels. Its partner institutions have strong advantages in the field of energy research, such as Chongqing University. Moreover, the burst time of the Hong Kong Polytechnic University lasted for more than 10 years. Scholars such as Yu have conducted long-term research in this field and are the main contributors to this field at this institution. The burst value of North China Electric Power University is the highest, and J. J. Wang contributed the most publications. The bursts of Central South University and the University of Moratuwa started in 2018; these universities are rising stars in the field of hotel energy research.

The results of the authors' cooperation analysis indicate an insufficient number of close and long-term author collaboration networks globally. The existing author collaboration networks are relatively simple and small. The co-authorship of the productive authors has remained relatively fixed. Generally, a relationship exists between productive authors and burst authors. It can be concluded that the cooperation between high-yield authors has improved their burst values.

The results of the citation path analysis demonstrate that scholars with different types of expertise have performed collaborative research in recent years. Disciplines such as hotel management and tourism management have been integrated with energy, architecture, and environmental research, promoting the integrated development of hotel energy research.

## 4 Conclusions

Bibliometrics and visualization were used to analyze the cooperation among countries, institutions, and authors in the field of hotel energy research, and the key citation paths of core papers were determined. Countries and institutions with particular research strengths and productive authors were identified. In addition, the current status of research collaborations was analyzed at the macro, meso, and micro levels. Furthermore, this paper revealed the frontiers and mainstream aspects of research to predict possible trends of future hotel energy studies. The results provide researchers with information on hotel energy research and a reference for institutions aiming for collaboration in this field.

It was found that research collaborations were more common among institutions or countries located in close proximity, and collaborations between institutions or authors in similar research fields were common. However, interdisciplinary or cross-unit cooperation has emerged, promoting interdisciplinary research. Furthermore, the cooperation mode reveals that interdisciplinary research will become the mainstream of hotel energy research in the future. Therefore, it is necessary for countries worldwide to pursue interdisciplinary, *trans*-regional, and transnational cooperation to promote research collaboration among different disciplines. In addition, it is also essential to strengthen exchanges and cooperation among universities, institutes, and enterprises.

The results obtained by this bibliometric analysis are relatively objective and can provide reference and guidance for research management and the formulation of related policies. Moreover, this method can be used to predict the future trend of research. Most importantly, bibliometric methods enable scholars engaged in interdisciplinary research to enter new fields rapidly.

However, this paper has certain limitations. We only used the WoS database, resulting in potential omissions of valid information contained in other databases. The reason was that the HistCite software only supports data from the WoS database. Thus, it is possible that pioneering papers are not mapped in the citation network. In addition, it is not suitable to use the frequency of publication for recently published papers. Therefore, some key papers published in recent years may not be included in the citation network.

Since this paper only discusses hotel energy studies from the perspective of research cooperation, the distribution and evolution of the hotspots and frontiers of knowledge in this field have not been analyzed in-depth. Therefore, it is necessary to conduct a more detailed bibliometrics analysis on keywords and reference co-citations in follow-up studies.

## Funding

Not applicable.

## Author contribution statement

All authors listed have significantly contributed to the development and the writing of this article.

## Data availability statement

Data will be made available on request.

## Declaration of competing interest

The authors declare that they have no known competing financial interests or personal relationships that could have appeared to influence the work reported in this paper.
